# Case report: recurrent chest pain as initial manifestation of rapidly progressing light-chain cardiac amyloidosis with microvascular infiltration—a novel red flag associated with poor outcomes

**DOI:** 10.1093/ehjcr/ytaf521

**Published:** 2025-10-11

**Authors:** Carla Indennidate, Aldostefano Porcari, Rossana Bussani, Marco Merlo, Gianfranco Sinagra

**Affiliations:** Center for Diagnosis and Treatment of Cardiomyopathies, Cardiovascular Department, Azienda Sanitaria Universitaria Giuliano-Isontina (ASUGI), University of Trieste, Via Valdoni 7, 34149 Trieste, Italy; European Reference Network for Rare, Low Prevalence and Complex Diseases of the Heart (ERN GUARD-Heart), Via Valdoni 7, 34149 Trieste, Italy; Center for Diagnosis and Treatment of Cardiomyopathies, Cardiovascular Department, Azienda Sanitaria Universitaria Giuliano-Isontina (ASUGI), University of Trieste, Via Valdoni 7, 34149 Trieste, Italy; European Reference Network for Rare, Low Prevalence and Complex Diseases of the Heart (ERN GUARD-Heart), Via Valdoni 7, 34149 Trieste, Italy; Division of Medicine, National Amyloidosis Centre, University College London, Royal Free Hospital, Pond Street, London NW3 2PF, United Kingdom; Institute of Pathological Anatomy and Histology, Azienda Sanitaria Universitaria Giuliano-Isontina (ASUGI), University of Trieste, Via Valdoni 7, 34149 Trieste, Italy; Department of Medical, Surgical and Health Sciences, University of Trieste, Piazzale Europa 1, 34127 Trieste, Italy; Center for Diagnosis and Treatment of Cardiomyopathies, Cardiovascular Department, Azienda Sanitaria Universitaria Giuliano-Isontina (ASUGI), University of Trieste, Via Valdoni 7, 34149 Trieste, Italy; European Reference Network for Rare, Low Prevalence and Complex Diseases of the Heart (ERN GUARD-Heart), Via Valdoni 7, 34149 Trieste, Italy; Center for Diagnosis and Treatment of Cardiomyopathies, Cardiovascular Department, Azienda Sanitaria Universitaria Giuliano-Isontina (ASUGI), University of Trieste, Via Valdoni 7, 34149 Trieste, Italy; European Reference Network for Rare, Low Prevalence and Complex Diseases of the Heart (ERN GUARD-Heart), Via Valdoni 7, 34149 Trieste, Italy

**Keywords:** Microvascular infiltration, Microvascular dysfunction, Amyloidosis, Recurrent angina, Fatal cardiac amyloidosis, Case report

## Abstract

**Background:**

Angina due to microvascular amyloid infiltration is an under-recognized early red flag of infiltrative disease and should be investigated as a predictor of poor outcomes.

**Case summary:**

A 73-year-old man with cardiovascular risk factors and a history of treated obstructive coronary artery disease presented 10 months later with recurrent angina. His condition progressively worsened, with the development of severe dyspnoea. Within 6 months, he succumbed to cardiogenic shock in the presence of severe biventricular dysfunction and unobstructed coronary arteries. Post-mortem diagnosis revealed AL cardiac amyloidosis with massive myocardial infiltration and extensive microvascular amyloid deposits.

**Discussion:**

This case underscores chest pain as a critical clinical marker, potentially indicating microvascular amyloid infiltration in patients with light-chain cardiac amyloidosis, particularly those with unobstructed coronary arteries. Its assessment may also have implications for future therapies.

Learning points

- This case underscores chest pain as a critical clinical marker, potentially indicating microvascular amyloid infiltration in patients with light-chain cardiac amyloidosis (AL-CA), particularly those with unobstructed coronary arteries.
- Microvascular amyloid infiltration should be recognized as a key determinant of poorer outcomes and rapid progression in AL-CA, offering a potential target for emerging therapies designed to remove amyloid fibrils from the heart.

## Introduction

Systemic immunoglobulin light-chain (AL) amyloidosis is characterized by the production of misfolded immunoglobulin light chains by an abnormal clone of plasma cells that subsequently deposits within the interstitial space of multiple organs, resulting in progressive organ dysfunction.^1^ Cardiac infiltration [light-chain cardiac amyloidosis (AL-CA)] is present in up to 80% of patients and is the main driver for mortality.^2^ Median survival ranges from 2 months to 4 years depending on cardiac disease severity,^3^ which is usually assessed with the 2004 Mayo staging system with the 2013 European modification.^4,5^

Although patients often seek medical attention for the development of signs and symptoms of heart failure (HF), increased left ventricle (LV) wall thickness, and systolic dysfunction,^3,4^ chest pain is also a common feature, reported in up to one-quarter of cases,^6^ and can sometimes resemble acute coronary syndromes.^7,8^ Vascular and perivascular amyloid deposition is an under-recognized cause of myocardial ischaemia, potentially leading to rapid clinical deterioration and death.

We report a case of a patient with AL-CA who presented with chest pain and experienced rapid disease progression associated with microvascular amyloid infiltration, resulting in an adverse outcome.

## Summary figure

Timeline of the case vignette from the first clinical presentation to the last assessment. Angina pectoris resulting from microvascular ischaemia may serve as an initial indicator of light-chain cardiac amyloidosis. It is important to consider the role of microvascular amyloid infiltration in the rapid progression of the disease and the poor prognosis associated with it.

**Figure:**
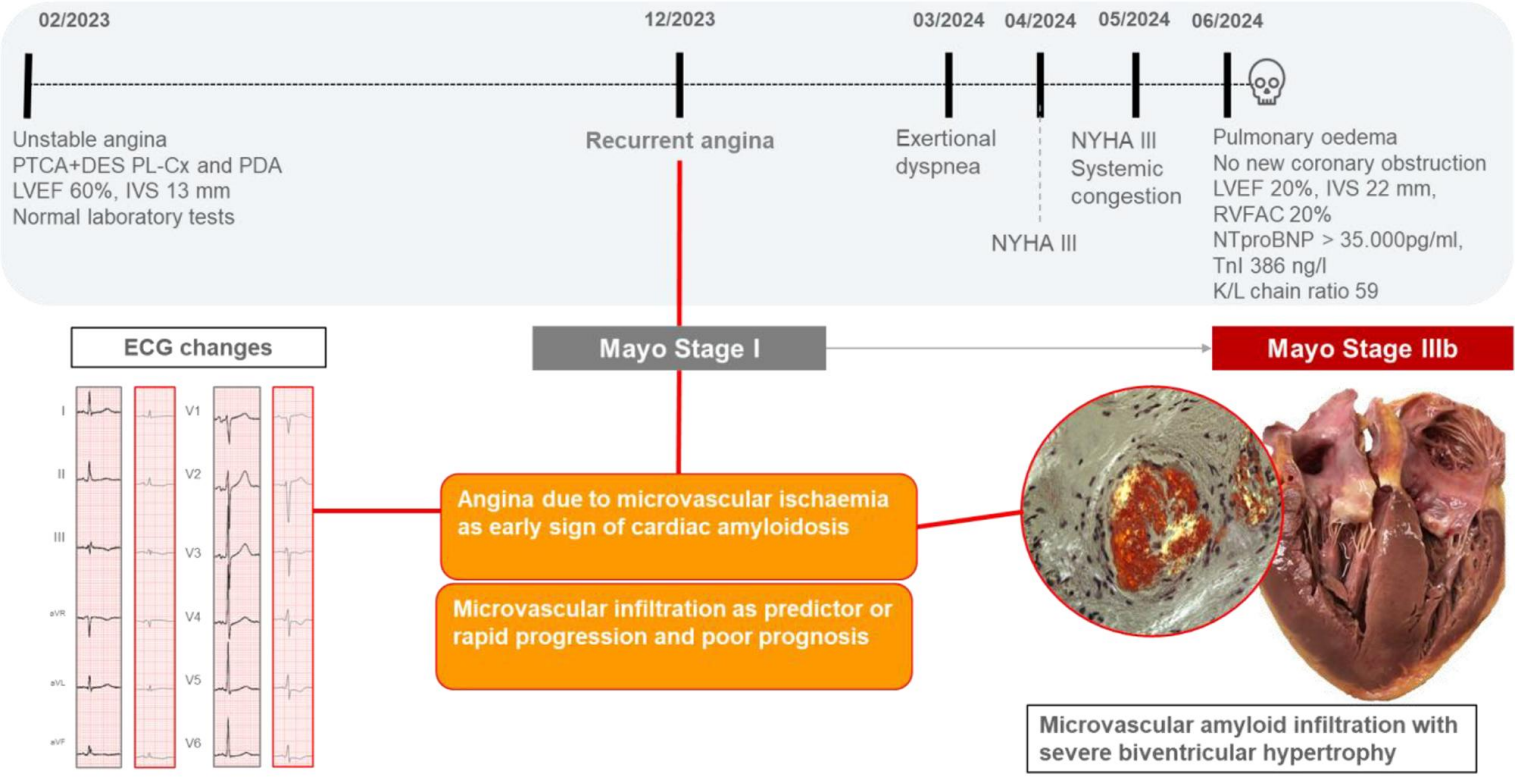


## Case presentation

**Figure 1:**
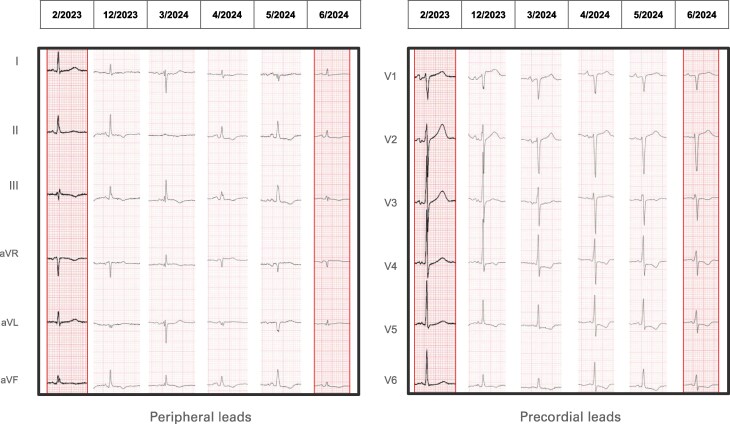
Series of electrocardiograms demonstrating dynamic changes during the disease course. The peripheral leads from February 2023 demonstrated left atrial enlargement, left ventricular hypertrophy, and residual subepicardial ischaemia in the inferior leads. In December 2023, the emergence of new lateral repolarization abnormalities is discernible, accompanied by a less conspicuous decline in peripheral voltages. From April 2024, it can be observed that there is fragmentation in lead aVL and a marked progressive reduction of peripheral voltages, to the extent that in June 2024, the QRS complexes are difficult to discern (<0.5 mV). The precordial leads demonstrate a progressive evolution of anterior pseudonecrosis, commencing in April 2024 and accompanied by the persistence of lateral negative T waves.

**Figure 2:**
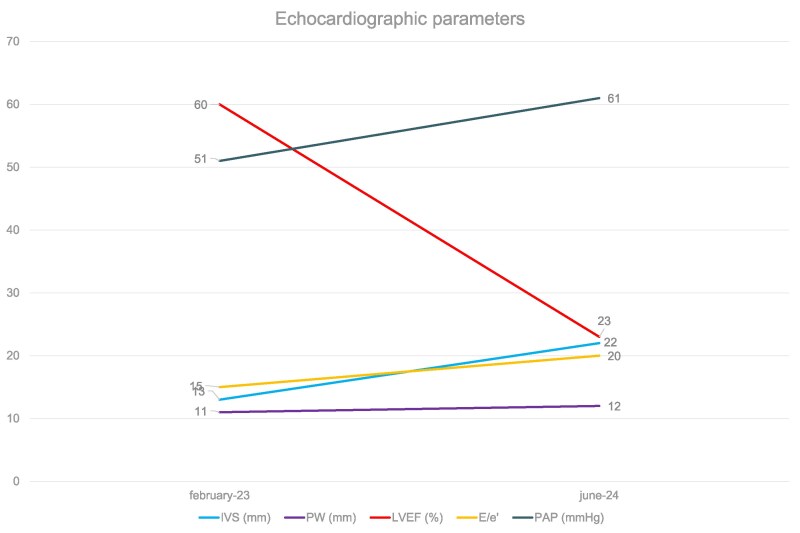
Change in key echocardiographic parameters over time. Echocardiographic findings. In February 2023, the patient was discharged with normal biventricular function and Grade 2 diastolic dysfunction. The well-known evolution of AL-CA is severe hypertrophy, which can lead to heart failure with preserved ejection fraction. However, in this case, the restrictive filling pattern was associated with severe biventricular dysfunction.

A 73-year-old Caucasian man with multiple cardiovascular risk factors (smoking, hypertension, dyslipidaemia, and type II diabetes mellitus), previous lung surgery for pulmonary plasmacytoma, and no cardiovascular history, presented in February 2023 with unstable angina (i.e. negative troponin I values). Coronary angiogram identified obstructive coronary artery disease of the circumflex artery’s posterolateral branch and the posterior descending artery, which was treated with stent placement. Residual mild stenosis on the proximal descending coronary artery and critical stenosis of small segments of the distal descending coronary artery, intermediate artery, first obtuse marginal artery, and small non-dominant proximal right coronary artery were left (see Supplementary material, Supplementary material online, *Videos S1* and *S2*). The electrocardiogram (ECG, *Figure 1*) showed sinus rhythm, heart rate 81 b.p.m., left atrial enlargement, LV hypertrophy, and ST segment/T wave changes in the inferior leads. Echocardiogram demonstrated normal LV dimension [end-diastolic diameter (EDD) 53 mm, end-diastolic volume indexed (EDVi) 49 ml/m^2^], slightly increased wall thickness (max 13 mm), type II diastolic dysfunction, *E*/*e*′ 15, mildly dilated left atrium and pulmonary artery systolic pressure 53 mmHg, left ventricle ejection fraction (LVEF) 60% in the absence of regional wall motion abnormalities (*Figure 2*, Supplementary material, Supplementary material online, *Video S3*), normal right ventricle (RV) dimensions, and systolic function (TAPSE 25 mm). Renal and thyroid function and serum electrophoresis were normal (eGFR 90 ml/min), with a near-normal NT-proBNP (243 pg/ml—reference normal values <300 pg/ml). The patient was discharged on a full anti-ischaemic regimen and aggressive antihypertensive therapy.

He remained asymptomatic until December 2023, when recurrent angina and poorly controlled blood pressure were reported along with new lateral repolarization changes on ECG. In March 2024, he developed exertional dyspnoea [New York Heart Association (NYHA) II] and mild peripheral oedema, but ECG, echocardiography, and blood tests were unchanged. In April 2024, he presented to the emergency department (ED) with dyspnoea on minimal exertion (NYHA III) with relevant peripheral oedema, suggesting heart failure with preserved ejection fraction (EF) and requiring initiation of diuretic therapy. ECG showed anterior ST/T wave changes, poor R wave progression in the precordial leads (a possible sign of anterior pseudonecrosis) and reduced peripheral voltages, with a slight increase in troponin (TnI: 99 ng/L—reference normal values <22 ± 4 ng/L), reduced renal function (eGFR 64 ml/min), and elevation in liver tests (sGOT/sGPT: 95/83 IU/L—reference normal values <45 U/L). Chest X-ray revealed pulmonary congestion and cardiomegaly. The following month, the patient required diuretic intensification for sustained NYHA class III symptoms. Although the echocardiogram, performed at another hospital, was reported as unchanged, ECG demonstrated a left posterior fascicular block, with more pronounced septal necrosis and persistent fragmentation in lead aVL, and unchanged peripheral low voltages. Blood tests showed persistent liver damage (SGOT/SGPT: 43/75 IU). On 5 June 2024, he was admitted to the ED with pulmonary oedema. Echocardiogram revealed severely increased wall thickness (max 22 mm) with normal-sized chambers (EDD 50 mm, EDVi 46 ml/m2), severe biventricular dysfunction (LVEF 23%, TAPSE 16 mm), restrictive diastolic pattern (*E*/*e*′ 20, systolic pulmonary arterial pressure 61 mmHg) (*Figure 2*, Supplementary material, Supplementary material online, *Videos S4* and *S5*). NT-proBNP was above 35 000 pg/ml with an eGFR of 50 ml/min, elevated troponin I (TnI: 386 ng/L), and monoclonal gammopathy with significant kappa free light chains excess (IgG kappa 1242 mg/L, reference normal values 3.30–19.40 mg/L; lambda 20 mg/L, reference normal values 5.71–26.30 mg/L; K/L ratio 59.39, reference normal value 0.26–1.65), which increased the suspect of AL-CA although no other signs of amyloidosis could be detected. Repeated invasive angiogram demonstrated unobstructed disease (see Supplementary material, Supplementary material online, *Videos S6* and *S7*). The patient developed fast atrial fibrillation leading to haemodynamic deterioration despite inotropic support. Multiple cardioversion attempts were made, but the patient died on June 10th due to refractory ventricular fibrillation and pulseless electrical activity.

**Figure 3:**
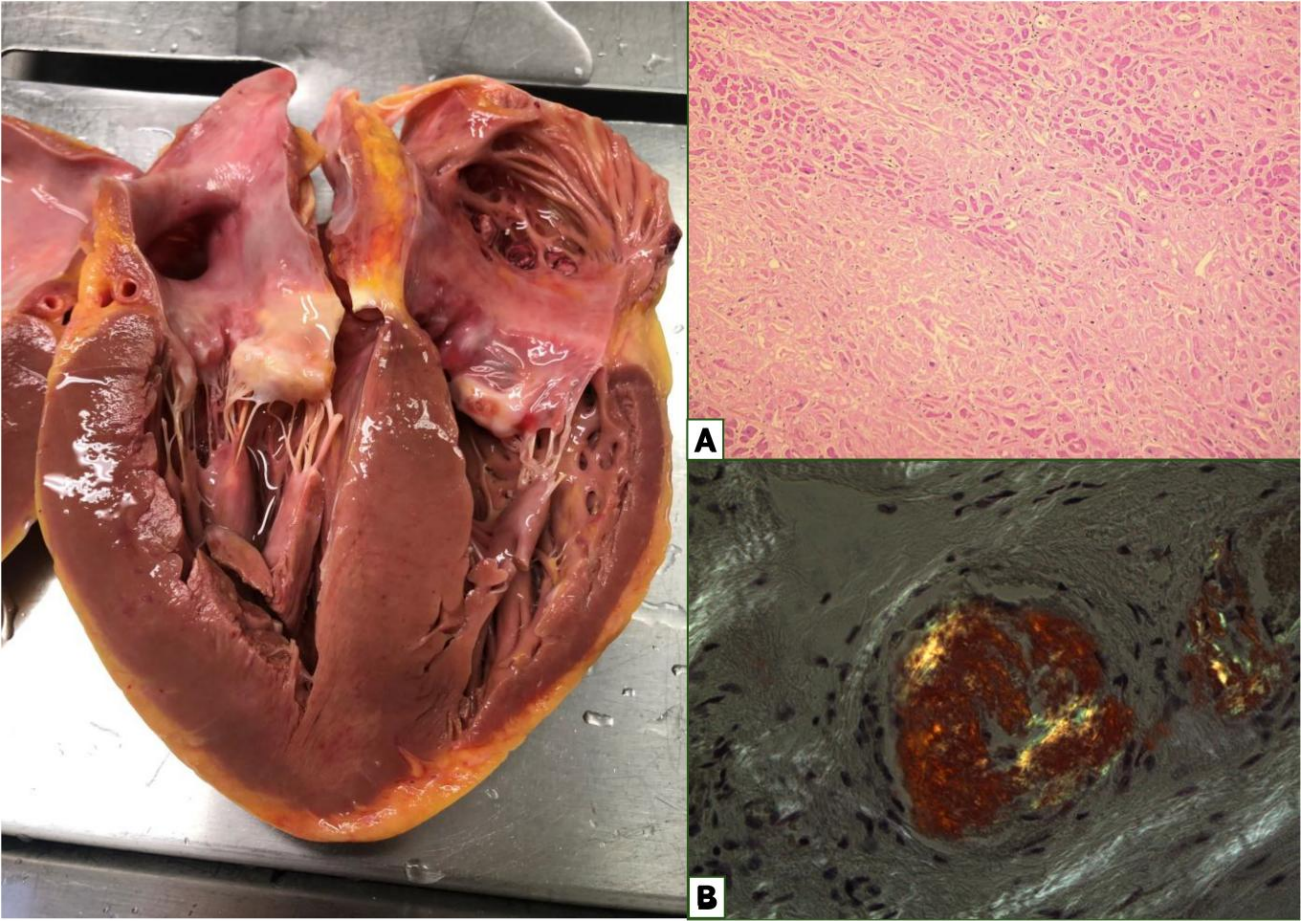
On the left, gross evaluation of unfixed heart following longitudinal dissection. Diffuse increase in wall thickness in both ventricles, in the interventricular septum involving the subvalvular aortic region, extending to the papillary muscles, atrioventricular valves, the interatrial septum, and both atria. Overall heart weight was 620 g. The heart exhibits a heterogeneous colour with pale areas and a faint yellowish tone, suggesting myocardial ischaemia. On the right, histological evaluation of myocardial samples. (*A*) Haematoxylin and eosin staining, original magnification 40×: a substantial interstitial infiltration is evident in the myocardial tissue, resulting in disruption of the myocardial structure and marked capillary rarefaction. (*B*) Congo red staining under polarized light microscopy, original magnification 100×: the image demonstrates the presence of relevant microvascular cardiac infiltration, which has resulted in critical stenosis of a small arteriole. The sample demonstrated evidence of secondary myocardial ischaemia. Additionally, amyloid deposits were observed in the pulmonary, hepatic, splenic, and renal micro- and medium-sized arteries.

Post-mortem examination revealed a definitive diagnosis of AL-CA: severe cardiomegaly (heart weight, 620 g; *Figure 3*) with massive amyloid infiltration involving both ventricles, septum, valves, and atria. There was extensive microvascular amyloid infiltration resulting in critical narrowing of small and medium arterioles, ultimately leading to significant myocardial ischaemia (*Figure 3A* and *B*). Amyloid deposits were also found in the pulmonary, hepatic, splenic, and renal arteries. Hepatosplenomegaly and severe pulmonary haemorrhagic oedema were noted.

## Discussion

We reported an uncommon case of a patient with AL-CA and extensive microvascular amyloid infiltration presenting with chest pain who experienced rapid disease progression. While reports of rapidly progressing HF in AL-CA are limited, available data underscore the central role of diastolic dysfunction as a key driver of symptoms and HF progression.

The case presented is unique. The patient initially appeared to have angina with critical coronary artery disease, a classic presentation of ischaemic heart disease, and was treated with stent placement. At the time of diagnosis, there were no cardiac or extracardiac findings to suggest amyloidosis. Chest pain is an uncommon and non-suggestive symptom of cardiac amyloidosis, especially when associated with critical coronary artery disease. However, the recurrence of chest pain in the absence of new epicardial coronary obstructions was a key feature in the patient’s clinical course. The second key feature was worsening HF associated with the evolution of ECG changes (*Figure 1*), with progressive reduction in QRS voltages and the development of conduction abnormalities. It was only a few days before the patient’s death that the clinical picture became suggestive of amyloidosis, with the diagnostic clue of diffuse ventricular wall thickening which was discordant with the low QRS voltages. This finding, corroborated by the presence of a serum monoclonal IgG kappa paraprotein, pointed to a diagnosis of AL-CA, later confirmed post-mortem.

The disease progressed dramatically within <6 months, with the patient presenting at first with Mayo Stage I and then rapidly evolving to Stage IIIB (elevated NT-proBNP, troponin, and dFLC levels) (*Table 1*). The delay in diagnosis prevented timely initiation of chemotherapy, which could have provided the greatest benefit. Although the haematologic efficacy of daratumumab-based schemes of treatment is very high, with 78% of patients achieving very good partial response (VGPR) or better,^1^ achieving early VGPR or better may still not ensure improved survival in Mayo Stage IIIB patients (deaths at 3 and 6 months, 18% and 43%, respectively).^9^

**Table 1:** Mayo stage and its components over time

	February 2023	June 2024
hs troponin I *(ng/L)*	18	386
NT-proBNP *(pg/ml)*	243	>35 000
dFLC *(mg/L)*	absent	1222
Mayo stage	I	IIIB

Disease progression in AL-CA is typically influenced by the extent of cardiac amyloid deposition^10^ and the cardiotoxic effect that light chains exert on the cardiomyocytes through proteome remodelling, activation of oxidative stress pathways, and induction of apoptosis.^11^ While elevated cardiac biomarkers in cardiac amyloidosis may also indicate microvascular infiltration alongside myocardial infiltration,^12,13^ cardiac magnetic resonance has the ability to provide key information on both extracellular volume and microvascular perfusion reserve at the patient level. In the presence of unexplained angina followed by dyspnoea with suggestive ECG changes, patients with AL amyloidosis should be comprehensively assessed for microvascular dysfunction as it is associated with poorer outcomes.^6,14^

We believe that microvascular amyloid infiltration was a key driver of mortality in this case. Amyloid deposition in the myocardial extracellular space disrupts myocardial structure and function but also infiltrates the vascular wall and disrupts the capillary architecture, ultimately leading to myocardial ischaemia and poor prognosis.^15,16^ However, microvascular amyloid infiltration is a rare and often underestimated finding, typically only identified at post-mortem examination. While most studies on this subject have relied on clinical, laboratory, and imaging data, they often lack final confirmation through histology. Our study is unique in that it combines clinical data, ECG changes, laboratory results, cardiac imaging, and histological evidence of extensive microvascular amyloid infiltration in the coronary vessels^14,15,17^ (*Summary figure*). Microvascular amyloid infiltration should be considered among the potential causes of rapid deterioration in patients with AL-CA and should prompt closer monitoring. Patients with AL-CA and microvascular amyloid infiltration might potentially benefit from antibody treatment to promote the clearance of amyloid deposits from vessels.

## Conclusion

Angina recurrence in the absence of narrowed epicardial coronaries may point towards amyloid infiltration in patients with dynamic ECG changes and clinical deterioration, representing a novel red flag of cardiac amyloidosis and a marker of adverse outcomes. This case emphasizes the need for greater awareness and inclusion of indications about microvascular dysfunction in future cardiac amyloidosis guidelines. In addition, this observation may have important implications in the development of novel therapies designed to remove amyloid fibrils from the heart, which may potentially enable resolution of microvascular amyloid infiltration. This may facilitate cardiomyocyte recovery and improve survival.

## Supplementary Material

ytaf521_Supplementary_Data

## Data Availability

The data underlying this article are available in the article and in its online Supplementary material.

## Lead author biography

Dr Carla Indennidate earned her MD from Università Campus Bio-Medico and is completing her cardiology residency at Trieste University, specializing in the Center for Diagnosis and Treatment of Cardiomyopathies. Her clinical and research interests focus on heart failure, advanced cardiac imaging, and cardiomyopathies. She is dedicated to advancing innovative therapeutic strategies and improving outcomes for patients with cardiovascular diseases. Through her work, she aims to integrate cutting-edge research into clinical practice and contribute to the development of personalized care approaches for complex cardiac conditions.
